# Development and validation of an algorithm for laser application in wound treatment ^1^

**DOI:** 10.1590/1518-8345.1998.2955

**Published:** 2017-12-04

**Authors:** Diequison Rite da Cunha, Geraldo Magela Salomé, Marcelo Renato Massahud, Bruno Mendes, Lydia Masako Ferreira

**Affiliations:** 2 MSc, Adjunct Professor, Phisiotherapy Department, Centro Universitário de Formiga , Formiga, MG, Brasil; 3 PhD, Associate Professor, Universidade do Vale do Sapucaí, Pouso Alegre, MG, Brazil; 4 MSc, Adjunct Professor, Phisiotherapy Department, Universidade do Vale do Sapucaí, Pouso Alegre, MG, Brazil; 5 Pós-Doctoral degree, Full Professor, Plastic Surgery discipline, Universidade Federal de São Paulo, São Paulo, SP, Brazil

**Keywords:** Wounds and Injuries, Wound Healing, Lasers, Protocols, Algorithms

## Abstract

**Objective:**

To develop and validate an algorithm for laser wound therapy.

**Method:**

Methodological study and literature review. For the development of the algorithm, a review was performed in the Health Sciences databases of the past ten years. The algorithm evaluation was performed by 24 participants, nurses, physiotherapists, and physicians. For data analysis, the Cronbach’s alpha coefficient and the chi-square test for independence was used. The level of significance of the statistical test was established at 5% (p<0.05).

**Results:**

The professionals’ responses regarding the facility to read the algorithm indicated: 41.70%, great; 41.70%, good; 16.70%, regular. With regard the algorithm being sufficient for supporting decisions related to wound evaluation and wound cleaning, 87.5% said yes to both questions. Regarding the participants’ opinion that the algorithm contained enough information to support their decision regarding the choice of laser parameters, 91.7% said yes. The questionnaire presented reliability using the Cronbach’s alpha coefficient test (α = 0.962).

**Conclusion:**

The developed and validated algorithm showed reliability for evaluation, wound cleaning, and use of laser therapy in wounds.

## Introduction

A wound is any interruption in the continuity of the corporeal tissue. The causes are mainly related to trauma or triggered by a clinical condition, specifically by wound dehiscence, traumatic wounds, vasculogenic ulcers, and pressure injuries^1^. Skin wounds affect people at any age; to repair this damaged tissue, the body uses intrinsic, dynamic, organized, and extremely complex biological processes that can occur quickly when the clinical situation is favorable, and the extent and degree of tissue loss are minor. Nevertheless, chronic wound lead to problems that affect the individual’s life, generating a negative impact, such as: presence of pain, alterations in the perception of the self-image, self-esteem and in spirituality, negatively modifying quality of life, and contributing to occupational disability, causing awkwardness and embarrassment in social relationships ^2^ ^-^ ^5^.

Several types of adjunctive treatment for acute and chronic wounds are available. Many research studies highlight the use of laser, ultrasound and phytotherapeutics. The choice of the best treatment depends on intrinsic and extrinsic factors, dynamic processes, and the clinical situation, at each moment of the evolution of the wound *healing phases* ^(^ ^6^ ^-^ ^7^. Laser is currently one of the main resources used by health professionals for treatment of wounds; its effects are based on the proliferation of fibroblasts, osteoblasts and epithelial cells, as well as in the synthesis of collagen, which is fundamental for good healing ^(^ ^7^ ^-^ ^8^.

Professionals using laser therapy to treat wounds need to acquire knowledge on the best form of treatment, absolute and relative contraindications, physiological effects, complications, application techniques, and Brazilian biosafety standards. Thus, the professional can offer safe, topical treatment of lesions, obtaining benefits regarding the process of wound healing^9^. Technical and scientific knowledge can be acquired by means of classes, trainings, and scientific articles; practice should be follow clinical guidelines, protocols, and booklets and validated algorithms, based on clinical evidence.

The algorithms consist of a finite sequence of well-defined instructions, systematically performed, which are commonly used in healthcare. Having a complete view of the clinical process, these instruments are simple, direct and easy-to-access ^(10^, in addition to constituting an indispensable tool for standardization of techniques and quality management, and are an important part of organization processes, acting as a guide for decision-making. ^(^ ^10^.

The development of an algorithm for laser wound therapy is favorable, along with an application based on the proposed algorithm for evaluation, wound cleaning, laser therapy, and primary dressing on wounds. These instruments can support a more objective evaluation of the area’s characteristics, the choice of the technique for laser application, and facilitate the recording of the lesion’s characteristics, ensuring monitoring of the evolution of the wound and assessment of the laser therapy results. This provides several benefits to the wound healing process and to patient safety, as well as greater safety for the professional during the application of the treatment. The objective of this study was the development and validation of an algorithm for laser therapy for wound care.

## Method

This was a methodological study and review of the literature, conducted through the Professional Master’s Program in Applied Sciences in Health, *Faculty* of *Health Sciences Dr*. *José Antônio Garcia Coutinho*, Universidade do Vale do Sapucaí (Univas), Pouso Alegre, MG, Brazil. The study was approved by the Institutional Research Ethics Committee, under protocol No. 1,154,935.

The sample consisted of 24 professionals (nurses, physiotherapists, and physicians) with e-mails registered at Univas, at Universidade Federal de São Paulo (Unifesp), in São Paulo, and at the Isa Rodrigues de Souza Skin Injury Nursing Care Center/ School of Nursing Wenceslau Braz, in Itajubá, MG, Brazil.

The inclusion criteria were having completed an undergraduate course in Physiotherapy, Nursing or Medicine at least three years prior to the study, and having at least 12 months of experience in the wound treatment with the use of laser therapy.

The algorithm was developed after a literature review in the health sciences databases, including the Cochrane Library, Scientific Electronic Library Online (SciELO), Latin American and Caribbean Health Science Literature (LILACS), National Library of Medicine, USA (MEDLINE), International Nursing Index (INI), Cumulative Index to Nursing and Allied Health Literature (CINAHL), and the Capes (Coordination for Improvement of Higher Education Personnel) *Journals Portal.*

To select the publications for review, only primary studies, guidelines and protocols associated with the subject, available as full text, were included; there was no limit on year of publication. Chapters of books, theses, dissertations, monographs, technical reports, reference studies and articles that, after reading the abstract, were not aligned with the purpose of the study were excluded, as well as duplicate publications in the databases and virtual library.

Studies regarding the subject were identified with the descriptors “injury and injuries”, “cicatrization”, “protocols”, “laser”, “algorithms” and “mobile application”, in English and Portuguese. After extensive bibliographical research, articles describing laser application parameters in the treatment of wounds,^7^ ^-^ ^20^ and guidelines to be used in the development of the algorithm, were selected^7^ ^,^ ^21^ ^-^ ^29^.

The wound evaluation steps, including measurement, wound margin type, tissue type, exudate type and amount present,^30^ and signs of infection were analyzed in the first stage of the algorithm. The second step determined the procedures that precede laser therapy, which provided inclusion of suggestions for wound cleaning techniques, according to the type of tissue found (i.e., devitalized, granulation and epithelial tissue)^7^ ^,^ ^22^ ^-^ ^24^. In the third step, the aim was to propose suggestions for laser therapy parameters, as therapeutic actions according to the type of tissue and exudate identified in the lesion. The fourth step involved the proposal of primary dressings, determined according to the professional prescription and the standardized coverage by the institution.

The content validation of the algorithm^31^ was performed by a committee of professionals, who were contacted by email, and the study was presented by way of an invitational letter. The invited professionals had to click on a link to confirm participation in the study and then, automatically, the Terms of Free and Informed Consent Form was signed. The expert committee consisted of professionals who agreed to participate in the study, who had access to the algorithm for its assessment through an electronic questionnaire.

The electronic questionnaire was developed using HyperText Markup Language (HTML), JavaScript, Cascading Style Sheets (CSS) and Active Server Pages (ASP). The use of CSS enabled the provision of the questionnaire on different device types (for example, computers, tablets or mobile phones). The language chosen for integration with the database was ASP.

The responses to the questions were arranged in a four-point Likert scale (poor, average, good and excellent) related to the items: graphic presentation, readability, algorithm sequence, wound evaluation, wound cleaning, laser therapy, and primary wound dressing. The responses “excellent” and “good” were classified as positive, while “average” and “poor” responses were characterized as negative. To be considered applicable, the algorithm required at least 70% positive responses.

Finally, the participants answered questions whose options were dichotomous, “yes” and “no”, and were related to the algorithm’s ability to support professional decision-making. A space was available for comments or suggestions after each question. If positive responses were less than 70%, adjustments requested by the respondents were made, and the instrument was sent back to the experts for reassessment.

The data were tabulated electronically, using the Excel 2007 program (Microsoft Corporation, Redwood, WA, USA), and analyzed quantitatively. Statistical analysis was performed using the IBM SPSS Statistics program, version 20 (IBM Corp., Armonk, NY, USA). The Cronbach’s alpha coefficient (α> 0.70) evaluated the reliability of the questionnaire, ^32^ and the Chi-square test was used for associations between variables. The level of significance was established at 5% (p <0.05).

## Results

The participants’ ages ranged from 28 to 66 years, of which ten (41.7%) were ages 50-66 years, eight (33.3%) were 28 - 39 years, and six (25%) were 40 - 49 years. There were 14 nurses (58.4%), eight (33.3%) were physiotherapists, and two (8.3%) were physicians. The period of time after participants’ undergraduate completion ranged from four to 44 years. The majority of participants (n=23, 95.8%) had completed a graduate degree. There was no statistical difference found with regard to the variable, period of time after undergraduation (Table 1).

**Table 1:** Distribution of the study participants, according to academic background, period of time after undergraduate completion, and graduate degree. Pouso Alegre, MG, Brazil, 2016

**Variables**	N*	**%** ^**†**^	**Valid %** ^**†**^	**Accumulated %** ^**†**^
**Academic education**				
**Nursing**	**14**	**58.4**	**58.4**	**58.4**
**Physiotherapy**	**8**	**33.3**	**33.3**	**91.7**
**Medicine**	**2**	**8.3**	**8.3**	**100**
**Total**	**24**	**100**	**100**	
**p-value** ^**‡**^		**0.023** ^**§**^		
**Period of time after undergraduate completion (years)**				
**4 - 9**	**5**	**20.8**	**20.8**	**20.8**
**10 - 19**	**6**	**25.0**	**25.0**	**45.8**
**20 - 29**	**7**	**29.2**	**29.2**	**75.0**
**30 - 44**	**6**	**25.0**	**25.0**	**100**
**Total**	**24**	**100**	**100**	
**p-value** ^**‡**^		**0.079** ^**§**^		
**Graduate degree**				
**Yes**	**23**	**95.8**	**95.8**	**95.8**
**No**	**1**	**4.2**	**4.2**	**100**
**Total**	**24**	**100**	**100**	
**p value** ^**‡**^		**0.003** ^**§**^		

* Population size; † Percentage; ‡ Descriptive level; § Significance level p <0.05 (chi-square test for independence)

**Figure 1:**
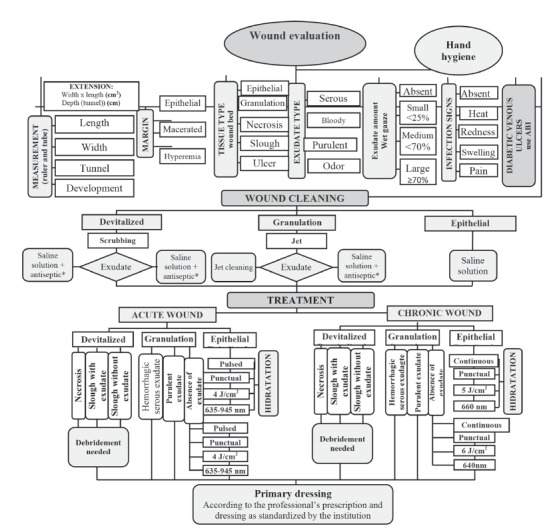
Algorithm for laser therapy in wounds. Pouso Alegre, MG, Brazil, 2016

The algorithm was developed based on the researched literature. Changes in the algorithm were performed after assessment by the expert committee, so that health professionals could evaluate the wound, define the type of cleaning, and the most appropriate parameters of laser therapy, for each type of tissue that could contribute to wound healing, as shown in Figure 1.

* The use of “saline solution” or “saline + antiseptic” in the presence of exudate may be considered in the “wound cleaning” stage, only in countries where standards for wound treatment do not include the use of antiseptics. The use of an antiseptic in this step is highly recommended.

**Figure 2:**
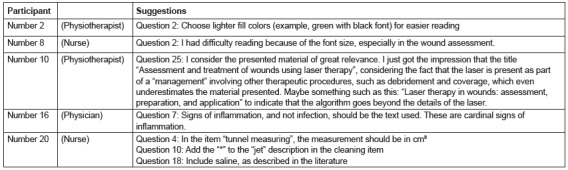
Synthesis of the qualitative analysis of participant suggestions that validated the algorithm. Pouso Alegre, MG, Brazil, 2016

The suggestions made by the experts are listed in Figure 2.

The professionals’ responses to the questions on the electronic questionnaire are presented in Table 3. No statistical difference was identified regarding the answers to the question about ease of reading the algorithm. The number of participants who chose positive responses (i.e., “excellent” and “good”) was significantly higher for all questions (Table 2).

**Table 2:** Evaluation of the algorithm by the participants. Pouso Alegre, MG, Brazil, 2016

**Questions**	**Poor**		**Average**		**Good**		**Excellent**		**p value** ^**‡**^
	n*	**%** ^**†**^	n*	**%** ^**†**^	**n** ^**†**^	**%** ^**§**^	**n** ^**†**^	%*	
**Graphic presentation**	**0**	**0**	**1**	**4.2**	**5**	**20.8**	**18**	**75.0**	**0.001** ^**§**^
**Ease of reading**	**0**	**0**	**4**	**16.7**	**10**	**41.7**	**10**	**41.7**	**0.856** ^**§**^
**Sequence of the algorithm**	**0**	**0**	**1**	**4.2**	**3**	**12.5**	**20**	**83.3**	**0.001** ^**§**^
**Description**									
**Wound measurement technique**	**0**	**0**	**2**	**8.3**	**5**	**20.8**	**17**	**70.8**	**0.001** ^**§**^
**Exudate type**	**0**	**0**	**4**	**16.7**	**3**	**12.5**	**17**	**70.8**	**0.001** ^**§**^
**Assessment of exudate amount**	**0**	**0**	**1**	**4.2**	**3**	**12.5**	**20**	**83.3**	**0.001** ^**§**^
**Signs of infection**	**1**	**4.2**	**1**	**4.2**	**3**	**12.5**	**19**	**79.2**	**0.001** ^**§**^
**Scrubbing cleaning technique for devitalized tissue**	**2**	**8.3**	**0**	**0**	**3**	**12.5**	**19**	**79.2**	**0.001** ^**§**^
**“Jet” cleaning technique for granulation fabric**	**1**	**4.2**	**1**	**4.2**	**5**	**20.8**	**17**	**70.8**	**0.001** ^**§**^
**Indication of ABI** ^**||**^	**0**	**0**	**2**	**8.3**	**5**	**20.8**	**17**	**70.8**	**0.001** ^**§**^
**Indication of cleaning technique for epithelial wound tissue**	**1**	**4.2**	**1**	**4.2**	**3**	**12.5**	**19**	**79.2**	**0.001** ^**§**^
**Therapeutic action on acute wound with devitalized tissue**	**1**	**4.2**	**3**	**12.5**	**5**	**20.8**	**15**	**62.5**	**0.007** ^**§**^
**Laser therapy parameters for acute wound with granulation tissue**	**1**	**4.2**	**1**	**4.2**	**7**	**29.2**	**15**	**62.5**	**0.007** ^**§**^
**Laser therapy parameters for acute wound with epithelial tissue**	**1**	**4.2**	**2**	**8.3**	**5**	**20.8**	**16**	**66.7**	**0.007** ^**§**^
**Therapeutic action on chronic wound with devitalized tissue**	**1**	**4.2**	**1**	**4.2**	**6**	**25.0**	**16**	**66.7**	**0.007** ^**§**^
**Laser therapy parameters for chronic wound with granulation tissue**	**1**	**4.2**	**2**	**8.3**	**5**	**20.8**	**16**	**66.7**	**0.007** ^**§**^
**Laser therapy parameters for chronic wound with epithelial tissue**	**1**	**4.2**	**2**	**8.3**	**3**	**12.5**	**18**	**75.0**	**0.001** ^**§**^

*Population size; †Percentage; ‡Descriptive level; §Level of significance p <0.05 (chi-square test for independence); ||Ankle-brachial index

**Table 3:** Cronbach’s alpha values and consistency of validation questionnaire questions Pouso Alegre, MG, Brazil, 2016

**Questions**	**Mean if item excluded**	**Variance if item excluded**	**Corrected item-item correlations**	**Cronbach’s alpha if item excluded**
**Graphic presentation**	**67.63**	**121.549**	**0.770**	0.958*
**Ease of reading**	**68.08**	**121.210**	**0.581**	0.960*
**Sequence of the algorithm**	**67.54**	**125.042**	**0.517**	0.961*
**Description**				
**Wound measurement technique**	**67.71**	**120.824**	**0.699**	0.959*
**Exudate type**	**67.79**	**116.607**	**0.831**	0.957*
**Assessment of exudate amount**	**67.54**	**121.911**	**0.802**	0.958*
**Signs of infection**	**67.67**	**124.145**	**0.381**	0.963*
**Scrubbing cleaning technique for devitalized tissue**	**67.71**	**116.911**	**0.714**	0.959*
**“Jet” cleaning technique for granulation fabric**	**67.75**	**118.804**	**0.697**	0.959*
**Indication of ABI** ^**||**^	**67.71**	**118.737**	**0.853**	0.957*
**Indication of cleaning technique for wound epithelial tissue**	**67.67**	**119.014**	**0.698**	0.959*
**Therapeutic action on acute wound with devitalized tissue**	**67.92**	**115.819**	**0.770**	0.958*
**Laser therapy parameters for acute wound with granulation tissue**	**67.83**	**118.145**	**0.733**	0.958*
**Laser therapy parameters for acute wound with epithelial tissue**	**67.83**	**115.101**	**0.860**	0.957*

*Level of significance α> 0.7 (Cronbach’s alpha coefficient); **||**Ankle-brachial index

In addition, 21 (87.5%) participants stated that the algorithm contained sufficient information to support decisions related to wound evaluation and cleanliness (p=0.001), and 22 (91.7%) stated that the information was sufficient to support decisions regarding the choice of laser parameters (p=0.001), with a statistically significant difference for all questions.

Table 3 shows that all the questions presented in the algorithm favorably contributed to the internal consistency of the instrument, as the score was 0.962. In the corrected item-item correlations, the question about the “Description of signs of infection” presented a weak correlation (0.381).

## Discussion

Professionals who provide care to wound patients need to be in a position to adapt to technological changes and globalization, which provide ideological, cultural and social changes in individuals. To successfully handle change, it is necessary to seek excellence in the profession. The accelerated increase of knowledge, and the volume of information generated, require a professional profile with learning capacity and rapid adaptation to the current context, developing skills and strategies to perform assistance based on scientific and technological evidence^21^.

In the present study, an algorithm was developed, which performs as a clinical tool for decision-making in laser wound treatment. The development of the algorithm was based on the scientific evidence found in the literature, and the knowledge and experience of professionals in the areas of physiotherapy, medicine and nursing, distributed as follows: 8.3% physicians, 33.3 % physiotherapists, and 58.4% nurses. Most of the participants had worked more than ten years since they had completed their undergraduate studies.

The labor market is becoming more competitive, leading professionals to progressively be more specialized to retain their jobs. By means of one specialization degree, the professional acquires technical and scientific knowledge, based on evidence ^10^ ^,^ ^13^. The use of protocols in the form of an algorithm, in the clinical environment, supports the systematic registration of care, allowing continuity of treatment and promoting quality of care. The systematic treatment of wounds minimizes healing time, and allows the analysis of costs and benefits of the treatment used^10^ ^,^ ^33^.

The choice of the subject, Algorithm for application of laser therapy in wound care: application development, arose from the difficulties of researchers in their care activities to find criteria for the application of lasers in wound treatment. It is known that laser therapy is a wound treatment, since it accelerates tissue proliferation, increases vascularity in the wound area, and the formation of more organized granulation tissue, favoring rapid healing of the wound. However, criteria for its application are necessary^5^ ^,^ ^8^ ^-^ ^9^ ^,^ ^14^.

In the present study, the proposed algorithm was evaluated by means of a questionnaire developed by the researchers, based on another study with the same purpose, published in the literature ^34^. Most participants’ responses were considered positive, with “excellent” and “good” responses. Regarding the assessment of the ability of the algorithm to support decisions in the evaluation, cleaning and laser parameters to be used, the participants found that the instrument was able to support professional decision-making.

The development of an algorithm for wound evaluation must be strongly based on the literature and clinical evidence, in order to provide technical, clinical, administrative, and financial support, always aiming to improve patient care and to obtain the best results for the institution ^33)^. After validation, the algorithm needed some changes, which are important to finalize the algorithm ^33^. These corrections contribute to better understanding, effectiveness and integration of the algorithm in the institution, allowing the professional to choose the most appropriate dressing for wound healing, resulting in patient safety and cost reduction ^33^. In a study whose objective was to develop an algorithm to support the nursing decision in wound dressing selection, according to the type of injury in children, 95.8% of the nurses considered it important to apply this instrument to support their decision-making for selection of the appropriate dressing in children with wounds ^(^ ^34^.

In the present study, the majority of the participants agreed with the applicability of the algorithm for use of laser therapy in the clinical practice; that is, they considered it to be an important tool, which contains information capable of supporting the professional’s decision-making regarding the treatment of wounds. The results were submitted to reliability analysis of the instrument. Cronbach’s alpha coefficient values ​​(α=0.962) demonstrated the internal consistency of the instrument.

Based on the results, the algorithm is capable of guiding professionals in decision-making for laser application in the treatment of wounds. The participants had the opportunity to critique the possible flaws in the algorithm, in order to improve it. The researchers carefully analyzed the critiques, and those indicated as relevant were accepted and are identified in the results section of this paper. Those suggestions that did not add to, or were not related to the proposal of the present study, as well as those that did not present clinical evidence, were discarded and, in turn, are not mentioned in this paper.

Protocols, algorithms, booklets, manuals, flowcharts, and guidelines are important tools for coping with the different problems in patient care and management within the health services. In studies validated by scientific evidence, guidelines have a technical, organizational, and political nature. They are also focused on the standardization of clinical, surgical, and preventive procedures ^7^ ^,^ ^10^ ^,^ ^33^ ^-^ ^34^. The development of new tools requires the incorporation of new technologies that meet the treatment needs, as well as the needs of the organizations that provide health care.

The algorithm collaborates with the use of laser therapy in wounds, offering theoretical and practical support to health professionals, and contributing to the standardization of evaluation, preparation, and application of laser therapy in wound treatment, resulting in improved patient care, individualized and systematized care, and higher safety for the health professional and for the patient.

Difficulties with having to consult procedure manuals are found in the clinical and academic environment, mainly by the professionals, primarily because the content is very theoretical, and the descriptions are very long. Thus, the proposal in this work was to facilitate the professionals’ access to information.

In this study, we had as a limitation of this study the lack of verification of the algorithm by means of its application during the clinical care.

## Conclusion

The developed and validated algorithm presented reliability for evaluation, cleaning, and the use of laser therapy in wounds, showing a viable basis for the application’s development.
